# Supplementary material for: Functional data analysis and visualisation of three‐dimensional surface shape
Source: J R Stat Soc Ser C Appl Stat. 2021 May 6;70(3):691–713. doi: 10.1111/rssc.12482 (PMC8518487; doi:10.1111/rssc.12482)
Supplement: Supplementary file 1 — Animated versions of some of the figures in the paper are available by downloading files from http://dx.doi.org/10.5525/gla.researchdata.1130. [file RSSC-70-691-s001.pdf]

6 Supplementary materials

Animated version of some of the Figures in the paper are available through the links below.

Figure 4:

http://www.stats.gla.ac.uk/~adrian/JCGS/FtoM-frontal.mp4

http://www.stats.gla.ac.uk/~adrian/JCGS/FtoM-lateral.mp4

Figure 6:

http://www.stats.gla.ac.uk/~adrian/JCGS/F-1-frontal.mp4

http://www.stats.gla.ac.uk/~adrian/JCGS/F-1-lateral.mp4

http://www.stats.gla.ac.uk/~adrian/JCGS/F-2-frontal.mp4

http://www.stats.gla.ac.uk/~adrian/JCGS/F-2-lateral.mp4

http://www.stats.gla.ac.uk/~adrian/JCGS/F-3-frontal.mp4

http://www.stats.gla.ac.uk/~adrian/JCGS/F-3-lateral.mp4

http://www.stats.gla.ac.uk/~adrian/JCGS/F-4-frontal.mp4

http://www.stats.gla.ac.uk/~adrian/JCGS/F-4-lateral.mp4

http://www.stats.gla.ac.uk/~adrian/JCGS/tour.mp4

Appendix A Surface curvature

The local shape at a 3D location $m = (x, y, z)$ on a differentiable manifold M can be characterised through the quadratic surface

$$z = \frac{1}{2}\left(\kappa_1(m)x^2 + \kappa_2(m)y^2\right),$$

where z lies in the normal direction to the surface at m and the orthogonal axes x and y lie on the tangent plane, associated with the directions of maximum, $\kappa_1(m)$, and minimum, $\kappa_2(m)$, curvature. This is clearly described by Koenderink (1990) and many others, along with a wide variety of other key tools for studying surface shape. The coefficients, $\kappa_1(m)$ and $\kappa_2(m)$, are referred to as the *principal curvatures* and, along with their associated *principal directions*, d_1 and d_2, they provide the essential information for characterising curvature across the manifold. A wealth of further detail is available in Koenderink (1990).

There is a variety of summary measures of curvature. An important one is *Gaussian curvature*, defined as the product of the principal curvatures, $\kappa_1(m)\kappa_2(m)$, which provides a summary of the size of curvature present at each location. Another particular quantity of interest is the *shape index*, which characterises the type of curvature present at each location. This is defined as

$$S(m) = \frac{2}{\pi}\tan^{-1}\left(\frac{\kappa_2(m) + \kappa_1(m)}{\kappa_2(m) - \kappa_1(m)}\right).$$

Koenderink and van Doorn (1992) provide further details.

Appendix B 3D warping

The technical details of warping are described here because the method is not widely used in 3D. We seek a function which maps X onto Y exactly. If an interpolant of a single co-ordinate of Y as a function of the three co-ordinates of X is considered, then the elegant functional analysis described by Duchon (1977) provides an immediate solution. The aim is to find the interpolating function f which has minimal bending energy, defined as

$$\int_{\mathcal{R}^3} \left\{ \sum_{p=1}^{3} \sum_{q=1}^{3} \left(\frac{\delta^2 f}{\delta x_p \delta x_q} \right)^2 \right\} dx_1 dx_2 dx_3.$$

The solution can be expressed in terms of radial basis functions which parameterise the relationship between points x and y in $\mathbb{R}^3$ as

$$y_d(x) = \sum_{j=1}^{J} \phi\left(||x - x_j||\right) \beta_{jd},$$

where β_{jd} are parameters and d denotes the three dimensions of $\mathbb{R}^3$. Fitting this functional form to the mapping from the observed locations in X to those in Y requires $Y = S\beta_1$, where S is a $J \times J$ matrix, with $S_{ij} = \phi\left(||x_i - x_j||\right)$, and β_1 is a $J \times 3$ matrix whose (j, d)th element is β_{jd}.

It is helpful to separate the mapping into affine and non-affine components, with the former capturing the linear part of the transformation, including possibly different scalings in different co-ordinate directions (shear), and the latter describing non-linear bending. If Q denotes the matrix $(1_J \; X)$, where 1_J is a column vector of 1's, then the transformation can be written

in the multivariate form

$$Y = S\beta_1 + Q\beta_2,$$

where β_2 is a 4×3 matrix filled with parameters. This system is now over-parametrised, with $(J+4) \times 3$ parameters but only $J \times 3$ defining equations. This can easily be resolved by adopting suitable constraints, for example through the extended system

$$\begin{pmatrix} Y \\ 0 \end{pmatrix} = \begin{pmatrix} S & Q \\ Q^T & 0 \end{pmatrix} \begin{pmatrix} \beta_1 \\ \beta_2 \end{pmatrix}, \tag{4}$$

where the 0 entries indicate matrices filled with 0's of the dimensionality required by the context. These constraints require the sum of the entries of each column of β_1 to be 0 and the sum weighted by the co-ordinates of each dimension of X also to be 0. By applying constraints to the affine component, the interpretation of the non-affine component is left undisturbed.

The system of equations (4) can be written in the condensed form $Y_e = X_e\beta$, with obvious definitions of X_e and Y_e. If the matrix S is invertible then so is X_e and, after some standard matrix manipulations, the solutions emerge as

$$\begin{aligned} B_e &= \left(S^{-1} - S^{-1}Q\left(Q^TS^{-1}Q\right)^{-1}Q^TS^{-1}\right), \\ \beta_1 &= B_eY, \\ \beta_2 &= \left(Q^TS^{-1}Q\right)^{-1}Q^TS^{-1}Y. \end{aligned}$$

When the *bending energy* matrix B_e is post-multiplied by X, this generates

the coefficients of the non-affine part of the transformation. The bending energy itself can be expressed as $\operatorname{tr}\left\{Y^T B_e Y\right\}$. Finally, the optimal radial basis function is shown simply to be $\phi(z) = -\frac{1}{8\pi} z$.
